# Reflections on Terri

**DOI:** 10.1101/gad.350472.123

**Published:** 2023-01-01

**Authors:** Karen Vousden

**Affiliations:** p53 and Metabolism Laboratory, the Francis Crick Institute, London NW1 1AT, United Kingdom

While a career in research can be one of the most interesting and rewarding of endeavors, it is marked by highs and lows that are often associated with getting the fruits of our labors published. I'm sure all scientists have had the experience of crushing disappointment when the reviewers of our work fail to grasp the significance of our study or identify defects that may be real but are more often (in our opinion) quite unfounded. The stress of publishing is greatly ameliorated by the ability to have a sensible discussion with an Editor who has a deep understanding of biology, impeccable judgement, and the confidence to make a decision. Over the years, there has been no better Editor of a scientific journal than Terri Grodzicker. At the helm, Terri ensured that *Genes & Development* became and remained a journal that publishes interesting, impactful, and sound science. Equally importantly, authors felt their papers were being handled by someone who understood and cared. She didn't always accept my papers (generally not) but was always fair, prompt, and incisive, and—very importantly—her advice inevitably made the study better. Of course, Terri never let standards slip, and over the years, *G&D* published some of the most important and influential papers in my field of p53. A review written by David Lane and Sam Benchimol in 1990 summarized the massive inflection point in our understanding of p53 biology that occurred at around this time. For almost 10 years, laboratories around the world had investigated the consequences of overexpressing p53 in cells, showing in numerous models that p53 expression could contribute to immortalization and transformation, functioning as an oncogene. However, toward the end of the 1980s, some cracks began to appear in this model. As described eloquently in the 1990 *G&D* review, the oncogenic forms of p53 that were being studied were, in fact, mutant versions of a wild-type protein whose normal function is to impede tumorigenic transformation. This was a remarkable watershed moment for the p53 field that prompted an avalanche of publications. Many of the most significant of these were published in *G&D* under Terri's careful stewardship. This is just a small example of the influence Terri and the journal have had on some of the most important areas of biological exploration.

While Terri is probably known to most people around the world as the Editor of *G&D*, many of us have also had the privilege of getting to know Terri in person. Cold Spring Harbor Laboratory meetings benefited from Terri's attendance at the talks and social events. Indeed, the afternoon soirees that Terri hosted at her house became one of the high points of the meetings, providing an opportunity to talk to other speakers in a relaxed environment. Catching up with Terri at various other meetings across the globe was also a great pleasure—I have fond memories of many happy dinners. Terri would often check in advance to make sure some of her friends would be at her selected meetings, so that these dinners could be carefully planned.

In her characteristic calm and quiet way, Terri has made a positive impact on scientific progress and the careers of many researchers. I feel we owe Terri a huge debt of thanks for her commitment to us—the scientific community—over the years. It's been a pleasure knowing her, working with her, and eating with her. I'm looking forward to tracking the next phase of her remarkable career.

